# The Gut–Immune Axis in Treated HIV Infection: From Mucosal Damage to Chronic Inflammation and Therapeutic Opportunities—A Clinician-Oriented Narrative Review

**DOI:** 10.3390/microorganisms14061229

**Published:** 2026-05-29

**Authors:** Thomas N. Nitsotolis, Stelios F. Assimakopoulos, Maria Lagadinou, Alexia Papalexandrou, Nikolaos Krikis, Marios Kourtidis, Eirini Christaki, Haralampos Milionis

**Affiliations:** 1 1st Department of Internal Medicine and Infectious Diseases, Faculty of Medicine, School of Health Sciences, University of Ioannina, 45500 Ioannina, Greece; nik1082n@gmail.com (N.K.); markourtidis@gmail.com (M.K.); eirini.christaki@uoi.gr (E.C.); 2 Department of Internal Medicine, Medical School, University of Patras, 26504 Patras, Greece; sassim@upatras.gr (S.F.A.); m_lagad2004@yahoo.gr (M.L.); 3 Ionian Nephrology Center, 18450 Piraeus, Greece; alexiapapalexandrou@gmail.com

**Keywords:** HIV infection, gut microbiome, gut–immune axis, microbial translocation, chronic immune activation, inflammaging, NLRP3 inflammasome, cellular senescence, trained immunity, non-AIDS events, biomarkers, antiretroviral therapy, intrinsic restriction factors, REPRIEVE, geroscience

## Abstract

Combined antiretroviral therapy (cART) has transformed HIV into a manageable chronic disease. However, people living with HIV (PLWH) experience a 16-year reduction in comorbidity-free life expectancy compared to HIV-negative individuals, driven by persistent chronic immune activation despite virological suppression. Serious non-AIDS events (SNAEs)—including cardiovascular disease, metabolic disorders, and malignancies—now represent the predominant cause of morbidity. This narrative review provides a clinician-oriented synthesis of immunopathophysiological mechanisms driving chronic inflammation in treated HIV infection, focusing on the gut–immune axis, restriction factors, trained immunity, biomarker-guided risk stratification, and therapeutic strategies. We searched PubMed/MEDLINE, Embase, and Web of Science through April 2026 using terms related to HIV chronic immune activation, gut-associated lymphoid tissue, microbial translocation, inflammaging, restriction factors, trained immunity, and biomarkers. This review followed the SANRA checklist. Irreversible destruction of gut-associated lymphoid tissue (GALT), intestinal barrier dysfunction, microbial translocation, maladaptive trained immunity, persistent myeloid activation with NLRP3 inflammasome signaling and cellular senescence, and viral reservoir persistence collectively perpetuate systemic inflammation. Biomarkers, including sCD14, IL-6, and suPAR, independently predict mortality but are not pathogen-specific. The REPRIEVE trial demonstrated a 36% reduction in cardiovascular risk with pitavastatin (HR 0.64, 95% CI 0.48–0.84), validating inflammation as a therapeutic target. Integration of early cART, statin therapy, optimal antiretroviral selection, and emerging strategies—including GLP-1 receptor agonists and gut-directed therapies—offers a practical framework for reducing inflammation-associated comorbidities in virologically suppressed PLWH.

## 1. Introduction

Human immunodeficiency virus (HIV) belongs to the *Lentivirus* genus of the *Retroviridae* family and causes acquired immunodeficiency syndrome (AIDS), characterized by progressive destruction of CD4+ T lymphocytes and consequent immunological collapse [1,2]. The introduction of combined antiretroviral therapy (cART) has revolutionized HIV management, transforming what was once a fatal disease into a chronic, manageable condition with near-normal life expectancy for adherent patients [3,4].

Despite these remarkable therapeutic advances, people living with HIV (PLWH) on suppressive cART continue to experience elevated morbidity and mortality compared to age-matched HIV-negative individuals [5]. This excess burden is mainly attributable to serious non-AIDS events (SNAEs), encompassing cardiovascular disease, metabolic dysfunction-associated steatotic liver disease (MASLD), chronic kidney disease, osteoporosis, neurocognitive disorders, and non-AIDS-defining malignancies [6,7]. The pathophysiological basis underlying these complications centers on persistent chronic immune activation and systemic inflammation—a phenomenon termed “inflammaging”—that persists despite successful virological suppression [8,9].

Critically, even when overall life expectancy approaches that of the general population, PLWH experience a significantly reduced comorbidity-free life expectancy. A large Kaiser Permanente cohort analysis demonstrated that PLWH have a 9-year shorter overall life expectancy and a 16-year shorter comorbidity-free life expectancy compared to age-matched HIV-negative individuals [10]. This substantial “healthspan gap” represents the actual burden of HIV-associated chronic inflammation and underscores the urgent need for interventions targeting residual immune activation beyond virological suppression.

Recent epidemiological data from the Antiretroviral Therapy Cohort Collaboration demonstrated that while survival has improved markedly since 1996, excess mortality persists among PLWH compared to the general population, with non-AIDS causes now accounting for an increasing proportion of deaths in high-income settings [11]. Cardiovascular disease represents the leading cause of non-AIDS mortality, with PLWH demonstrating a 1.5- to 2-fold increased risk of myocardial infarction compared to HIV-negative individuals, even after adjustment for traditional risk factors [12,13]. This excess cardiovascular risk is increasingly attributed to HIV-associated chronic inflammation rather than conventional risk factors alone.

Several recent reviews have addressed individual components of HIV-associated inflammation, including the mitochondrial–immune axis, immunometabolism, and cardiovascular implications [14,15,16]. However, no existing review integrates intrinsic antiviral restriction factors, the gut–immune axis (including recent insights into trained immunity), a validated biomarker panel for clinical risk stratification, and current therapeutic strategies—including lessons from the REPRIEVE mechanistic substudies—into a unified clinician-oriented framework. This review addresses this gap by synthesizing these interconnected pathways to provide actionable guidance for managing chronic inflammation in virologically suppressed PLWH.

### Aim and Methodology

This narrative review aims to provide a clinician-oriented synthesis of the immunopathophysiology that sustains chronic inflammation in virologically suppressed people living with HIV, with particular emphasis on the gut–immune axis, and to translate these mechanisms into evidence-based and emerging therapeutic strategies. We searched PubMed/MEDLINE, Embase, and Web of Science from January 2000 through April 2026 using combinations of the following terms: “HIV”, “chronic immune activation”, “inflammaging”, “gut-associated lymphoid tissue”, “microbial translocation”, “gut microbiome”, “intrinsic restriction factors”, “trained immunity”, “NLRP3 inflammasome”, “cellular senescence”, “biomarkers”, “serious non-AIDS events”, “statins”, “integrase strand transfer inhibitors”, “GLP-1 receptor agonists”, and “senolytics”. Priority was given to randomized controlled trials, prospective cohort studies, and mechanistic investigations published in high-impact journals, while large review articles were used only when no primary source adequately covered a topic. Because this is a narrative—not a systematic—review, broader review articles were consulted only to provide conceptual framing for rapidly evolving fields (e.g., trained immunity, geroscience, the gut mycobiome) and as a starting point for identifying primary references through citation tracing; every clinical estimate, biomarker hazard ratio, and trial result cited in the present manuscript is sourced from the original publication rather than from a secondary synthesis, and no inferential claim or therapeutic recommendation rests solely on a review article. Reference lists of pertinent articles and major conference proceedings (CROI, IAS, EACS) were hand-searched. Reporting followed the SANRA checklist for narrative reviews.

## 2. HIV-1 Accessory Proteins and Their Contribution to Chronic Inflammation

**Figure 1:**
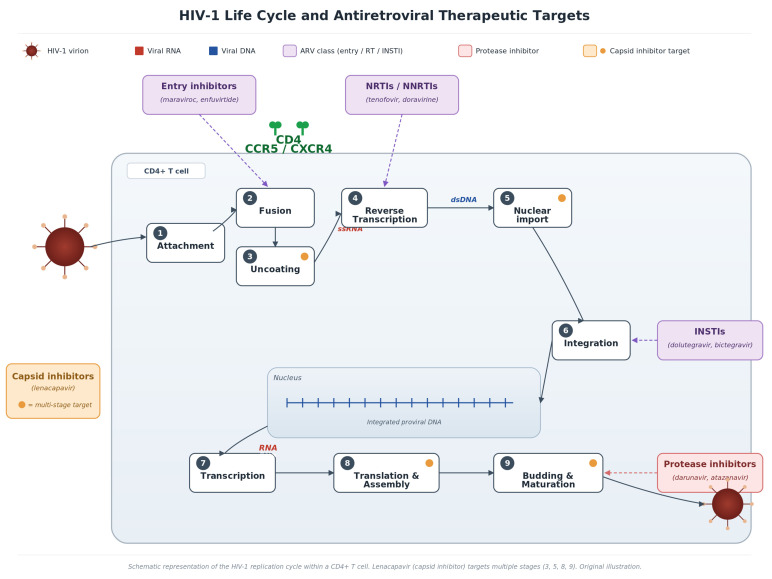
HIV-1 Life Cycle and Antiretroviral Therapeutic Targets. Schematic representation of the HIV-1 replication cycle within a CD4+ T cell, illustrating the nine stages from viral attachment to maturation. Purple boxes indicate major drug classes; the orange-red box highlights lenacapavir’s multi-stage targeting (steps 3, 5, 8, 9).

The HIV-1 genome comprises approximately 9.7 kilobases and encodes nine genes whose protein products are essential for structural, enzymatic, regulatory, and immune evasion functions [17,18]. While the structural genes (*gag*, *pol*, *env*) and regulatory genes (*tat*, *rev*) are well characterized and targeted by current antiretrovirals (Figure 1; Table S1), the four accessory genes—*nef*, *vif*, *vpr*, and *vpu*—are most directly relevant to persistent immune activation [19].

For the clinician, the practical point is that the four accessory proteins (Nef, Vif, Vpr, Vpu) do more than promote viral replication: they continue to fuel low-grade inflammation even when plasma viremia is undetectable. The detailed molecular functions of each accessory protein, with their viral counterparts and clinical relevance, are summarised in Table S1 and reviewed in depth elsewhere [19,20,21]. Briefly, Nef and Vpu turn off surface molecules that normally restrict virion release; Vif and Vpr disable host restriction factors and induce cell cycle perturbations; and the HIV-1 LTR couples viral transcription to T-cell activation, so any episode of immune activation can in turn reactivate residual provirus [22,23]. This circular relationship between accessory-protein activity and host immune activation explains why simply suppressing viremia with cART does not abolish the inflammatory drive—a concept that frames the rest of this review.

## 3. Intrinsic Restriction Factors and Chronic Immune Activation

Host cells express intrinsic antiviral restriction factors that provide cell-autonomous resistance against HIV-1 at multiple stages of the viral life cycle [24,25,26]. These factors—including APOBEC3G, SAMHD1, Tetherin, TRIM5α, MxB, and SERINC3/5—represent evolutionarily conserved defenses that HIV has partially overcome through viral antagonists (Table S2) [27,28,29,30,31]. The ongoing battle between restriction factors and their viral counterparts contributes to chronic immune activation even during suppressive cART [24,25,26].

APOBEC3G functions as a cytidine deaminase that introduces lethal G-to-A hypermutations in viral DNA during reverse transcription, effectively restricting HIV-1 replication [26]. HIV-1 counteracts APOBEC3G through the viral protein Vif, which targets APOBEC3G for proteasomal degradation [20,26]. SAMHD1 restricts HIV-1 in myeloid cells and resting CD4+ T cells by depleting the intracellular dNTP pool required for reverse transcription [31,32]. Tetherin (BST-2) physically retains budding virions on the cell surface, preventing viral release, and is counteracted by HIV-1 Vpu [21].

The cGAS–STING pathway represents a critical link between HIV sensing and systemic inflammation [33]. Recognition of incompletely reverse-transcribed viral DNA by cGAS triggers STING-dependent type I interferon production, which persists at low levels even during suppressive cART. This persistent innate immune activation drives the chronic inflammatory state underlying SNAEs and, importantly, primes monocytes for the epigenetic reprogramming characteristic of trained immunity (discussed in Section 4) [33,34]. TRIM5α restricts HIV-1 through premature capsid uncoating and has informed the development of capsid-targeting therapeutics, including lenacapavir [35]. MxB/MX2 blocks nuclear import of the pre-integration complex and synergizes with other interferon-stimulated genes [36,37].

## 4. Trained Immunity: Innate Immune Reprogramming in HIV

A paradigm-shifting concept in understanding persistent inflammation in treated HIV is trained immunity—the long-term functional reprogramming of innate immune cells through epigenetic and metabolic modifications [34,38]. Unlike adaptive immune memory, trained immunity confers a heightened, non-specific inflammatory response to subsequent stimuli, mediated by histone modifications (H3K4me3, H3K27ac) and metabolic rewiring toward glycolysis [38].

In the context of HIV, chronic exposure to microbial products—particularly β-glucan from *Candida* species that translocate across the disrupted gut barrier—and HIV-derived extracellular vesicles containing the Nef protein induce maladaptive trained immunity in monocytes [34,39]. van der Heijden et al. demonstrated that chronic HIV infection induces transcriptional and functional reprogramming of innate immune cells, characterized by enhanced priming of the IL-1β pathway and persistent production of proinflammatory cytokines [34]. Critically, this reprogramming persists during virological suppression: monocyte-derived macrophages from ART-suppressed individuals exhibit exaggerated cytokine responses (IL-6, TNF-α) to TLR agonists compared to HIV-negative controls [39].

This framework has profound implications for understanding and treating HIV-associated inflammation. First, it explains why microbial translocation (discussed in Section 6) has disproportionate inflammatory consequences in PLWH—innate immune cells are epigenetically primed to overreact to translocated microbial products. Second, it identifies novel therapeutic targets: IL-1β pathway blockade (e.g., canakinumab), epigenetic modifiers (e.g., HDAC inhibitors), and metabolic reprogramming (e.g., mTOR inhibitors) could reverse trained immunity and break the cycle of chronic inflammation [38,39]. The intersection of trained immunity with the gut–immune axis—where fungal translocation drives monocyte reprogramming—represents a frontier for therapeutic intervention.

A point that is often raised in the clinic deserves explicit comment: is “more” innate immune activity desirable in PLWH on suppressive cART? It should be acknowledged that “trained immunity” remains a conceptual framework derived largely from ex vivo and animal-model work; more cautiously phrased, what is operationally meant in PLWH is a set of persistent shifts in innate-immune reactivity. The maladaptive character of these shifts in treated HIV is supported less by direct in vivo demonstrations of epigenetic “training” than by consistent associations between elevated innate-immune markers (IL-6, sCD14, sCD163, IL-1β signatures) and somatic outcomes—cardiovascular events, frailty, and all-cause mortality—reported across independent cohorts and synthesized in earlier review work [38,40,41]. The available evidence indicates that, in this setting, persistent stimulation of innate immunity is predominantly maladaptive rather than protective. The trained-immunity programs induced by chronic exposure to translocated microbial products, residual HIV antigens, and CMV are dominated by IL-1β/IL-6/TNF-α priming and a senescence-associated secretory phenotype, and these very signatures correlate with cardiovascular events, frailty, and all-cause mortality in PLWH on ART [34,38,39]. In contrast, classical antimicrobial functions (e.g., phagocytic killing, vaccine responses) are often blunted rather than enhanced. The therapeutic implication, therefore, is to dampen the maladaptive (inflammatory) arm of trained immunity—via gut-barrier repair, optimized cART, statins, and selective cytokine or pathway blockade—rather than to broadly “boost” innate immunity.

## 5. Gut-Associated Lymphoid Tissue Destruction and Intestinal Barrier Dysfunction

### 5.1. Early Mucosal Immune Damage

The gastrointestinal tract represents the largest reservoir of lymphoid tissue and is the primary site of HIV-mediated immune destruction [42,43]. Within days of infection, massive depletion of CD4+ T cells occurs in the gut-associated lymphoid tissue (GALT), particularly affecting memory and effector CD4+ T cells expressing the gut-homing integrin α4β7 [44]. This early mucosal immune destruction is largely irreversible and persists despite effective cART, contributing to chronic intestinal inflammation and barrier dysfunction [45]. Th17 cells, which are essential for mucosal immunity and epithelial barrier maintenance through IL-22 production, are preferentially depleted in pathogenic lentiviral infections [45].

### 5.2. Mechanisms of Intestinal Barrier Disruption

HIV infection disrupts the intestinal epithelial barrier through multiple interconnected mechanisms: direct viral cytopathic effects on enterocytes, apoptosis induced by viral proteins (particularly gp120 and Tat), disruption of tight junction proteins by inflammatory cytokines, and loss of protective Th17 cells [46,47]. These changes increase intestinal permeability, leading to the translocation of microbial products into the systemic circulation.

A landmark 2025 study by Das Adhikari et al. elucidated a novel mechanism by which colon-resident CD8+ T cells directly contribute to epithelial barrier dysfunction in PLWH on suppressive ART [47]. Using patient-derived colonic organoids and murine models, the investigators demonstrated that CD8+ T cells in PLWH downregulate peroxisome proliferator-activated receptor-γ (PPARγ), thereby impairing fatty acid oxidation and reducing intracellular lipid droplets [47]. These metabolically dysregulated CD8+ T cells acquire lipids from adjacent intestinal epithelial cells through direct cell–cell contact, triggering epithelial apoptosis [47].

Critically, PPARγ agonists (thiazolidinediones) restored metabolic function and reduced epithelial damage in experimental models, identifying a potentially druggable pathway [47]. However, cardiovascular safety concerns and fracture risk associated with thiazolidinediones necessitate dedicated clinical trials in PLWH to establish the benefit–risk profile of this repurposing strategy [48,49].

### 5.3. Dysbiosis and Microbiome Alterations

**Figure 2:**
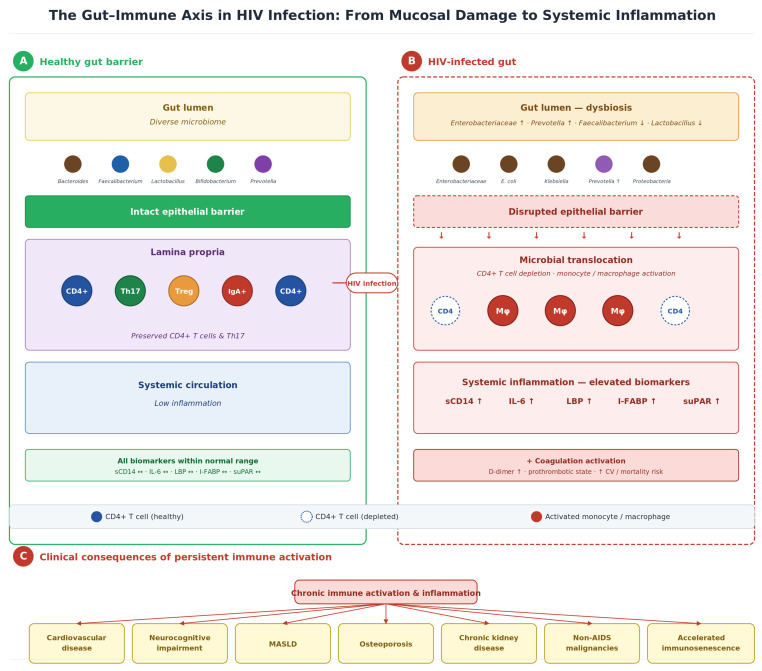
The gut–immune axis in HIV infection: from mucosal damage to systemic inflammation. Comparative illustration demonstrating (**A**) the healthy gut barrier with preserved CD4+ T cell populations and diverse microbiome, (**B**) HIV-induced mucosal damage with dysbiosis, microbial translocation, and monocyte/macrophage activation leading to elevated inflammatory biomarkers, and (**C**) clinical consequences of persistent immune activation, including cardiovascular disease, neurocognitive impairment, MASLD, osteoporosis, chronic kidney disease, and non-AIDS malignancies. Solid arrows indicate directional causal/mechanistic relationships within the gut–immune–systemic inflammation cascade; the curved feedback arrow denotes the bidirectional gut–systemic immune cross-talk. ↑ increase; ↓ decrease; ↔ normal.

HIV infection profoundly alters gut microbiome composition, characterized by decreased alpha-diversity, depletion of beneficial bacteria (*Lactobacillus*, *Bifidobacterium*, *Faecalibacterium*), and increased potentially pathogenic taxa (*Enterobacteriaceae*, *Prevotella*, *Proteobacteria*) [50,51,52,53]. Table S3 summarizes the key microbiome alterations observed in HIV infection and their clinical correlations. These changes persist despite virological suppression on cART, contributing to ongoing intestinal inflammation and barrier dysfunction (Figure 2).

At the genus level, a relatively consistent “HIV signature” has emerged across European, North American, and African cohorts: a shift away from *Bacteroides*-dominated enterotypes toward *Prevotella*-rich communities, expansion of *Proteobacteria*/*Enterobacteriaceae* (including *Escherichia coli* and *Klebsiella* spp.), and depletion of butyrate-producing *Firmicutes* such as *Faecalibacterium prausnitzii*, *Roseburia*, and *Eubacterium rectale* [50,51,52,54]. Two important caveats apply. First, part of the *Bacteroides*-to-*Prevotella* shift originally attributed to HIV is now recognized to reflect the MSM transmission risk group rather than HIV serostatus per se [55]. Second, the absolute pattern is geographically variable: Bashiardes et al. (2026), using shotgun metagenomics, recently confirmed an Israeli cohort enriched for *Prevotella* but identified expansion of *Escherichia coli* and *Klebsiella quasivariicola* as the dominant signature in an Ethiopian cohort, with the degree of dysbiosis tracking peripheral CD4+ T cell counts and modulating susceptibility to opportunistic pathogens such as *Cryptosporidium parvum* in fecal microbiota transplantation experiments [54]. Taken together, these heterogeneous but converging observations are consistent with the view that, in PLWH, gut dysbiosis is more than an epiphenomenon and may mechanistically contribute to systemic immune dysregulation and to susceptibility to opportunistic infections. We are nevertheless explicit that, given the cohort-to-cohort variability in the dominant taxa, the confounding by transmission risk group and geography, and the inherent difficulty of disentangling cause from consequence in cross-sectional data, a direct pathogenetic relationship should be inferred cautiously and ideally confirmed in longitudinal and interventional studies, including those using gnotobiotic or humanized-mouse models.

Notably, the gut mycobiome (fungal communities) is increasingly recognized as a driver of inflammation in PLWH [34,39]. *Candida* overgrowth following antibiotic exposure and immune dysregulation leads to β-glucan translocation, which directly triggers trained immunity in circulating monocytes via Dectin-1 receptor engagement, creating a mechanistic link between fungal dysbiosis and persistent systemic inflammation [34,39].

## 6. Microbial Translocation and Systemic Immune Activation

Microbial translocation refers to the passage of microbial products from the intestinal lumen across the compromised epithelial barrier into the systemic circulation [53]. This phenomenon was first demonstrated in chronic HIV infection by Brenchley et al., who identified elevated plasma levels of lipopolysaccharide (LPS) [53]. TLR4 engagement by LPS initiates MyD88- and TRIF-dependent signaling pathways, culminating in NF-κB and IRF3 activation and the subsequent production of proinflammatory cytokines including IL-6, TNF-α, and IL-1β [56]. In the context of trained immunity (Section 4), these translocated microbial products encounter epigenetically primed innate immune cells, amplifying the inflammatory response beyond what would be expected from microbial product levels alone.

Chronic inflammation does not remain confined to the cytokine network; it also engages the coagulation system. In practical terms, this means that sustained low-grade endotoxemia in PLWH translates into a measurable prothrombotic state, with elevated plasma D-dimer concentrations that—in cohort studies such as SMART, FRAM, and the VACS Index—independently predict all-cause mortality and incident cardiovascular events even after adjustment for traditional risk factors [57,58,59,60]. We interpret this not as an isolated coagulation phenomenon but as a clinically visible “readout” of the gut–inflammation axis, and it is part of the rationale for testing anti-inflammatory and gut-directed interventions in PLWH. Importantly, microbial translocation persists despite long-term virological suppression, indicating that cART alone is insufficient to restore gut barrier integrity or to normalize systemic immune activation [45,61].

## 7. Biomarkers of Immune Activation: Selection Rationale and Clinical Utility

### 7.1. Rationale for Biomarker Selection

Among the numerous biomarkers evaluated in HIV research, we focus on sCD14, IL-6, suPAR, I-FABP, and LBP based on three selection criteria: (1) mechanistic relevance to the gut–inflammation axis central to this review; (2) validated prognostic performance across ≥2 independent cohorts with multivariate adjustment for traditional risk factors; and (3) potential clinical applicability with commercially available assays. This focused panel directly reflects intestinal barrier damage (I-FABP), microbial translocation (LBP), monocyte/macrophage activation (sCD14), systemic inflammation (IL-6), and generalized immune activation (suPAR).

Several established biomarkers were not included: hs-CRP, while predictive of CVD in the general population, demonstrates weaker discrimination in PLWH and does not add incremental value beyond IL-6—a finding reinforced by the REPRIEVE mechanistic substudy showing non-significant hs-CRP reduction (*p* = 0.09) despite significant MACE reduction [62,63,64]; sCD163, although specific for M2 macrophage activation, has been primarily validated for neurocognitive outcomes rather than systemic SNAEs [65]; and neopterin, superseded mainly by suPAR with superior discriminative ability. D-dimer, reflecting coagulation activation downstream of inflammation, is included in Table 1 for completeness.

### 7.2. Biomarker Panel and Prognostic Value

Table 1 presents the selected biomarkers with their mechanisms, clinical associations, effect sizes, and key validating studies. The consistency of prognostic associations across SMART, ESPRIT, SILCAAT, and ACTG cohorts strengthens confidence in their clinical relevance [58,59,62,66,67,68,69,72].

An important interpretive caveat applies to these markers: none is HIV- or bacteria-specific. Chronic CMV reactivation (Section 8.2), HCV viremia before direct-acting antiviral cure, subclinical opportunistic infections, and even aging per se can raise sCD14, IL-6, suPAR, and D-dimer to levels comparable to those observed in patients with active disease [73,74,75,76]. Consequently, in clinical practice, a single biomarker measurement is not specific for pathogen translocation from the gut, and the panel should be interpreted in the context of the patient’s full virological, serological (CMV/HCV/HBV), and metabolic profile. The conceptual value of the panel is to flag patients with high inflammatory tone—regardless of the upstream driver—so that comprehensive risk-factor management (lipids, BP, lifestyle, statin therapy, treatment of coinfections) can be intensified.

Soluble CD14 (sCD14) is released by monocytes upon LPS stimulation and serves as a marker of monocyte activation in response to microbial translocation [66,77]. The SMART study demonstrated that elevated sCD14 independently predicted all-cause mortality with an odds ratio of 6.0 [66]. Interleukin-6 (IL-6), a pleiotropic proinflammatory cytokine, integrates multiple inflammatory signals and independently predicts mortality, cardiovascular events, and frailty [58,59,78,79,80]. The soluble urokinase plasminogen activator receptor (suPAR) reflects immune activation and predicts non-AIDS events during suppressive ART [67].

### 7.3. Clinical Utility and Implementation Considerations

While these biomarkers demonstrate robust prognostic value in research settings, their translation to routine clinical practice is hindered by implementation barriers. sCD14 and LBP are commercially available via ELISA (USD 50–150 per test), with sCD14 demonstrating superior discriminative ability (AUC 0.72–0.78) [66,69,70]. suPAR has the most developed commercial infrastructure (suPARnostic^®^ assay, CE-marked and FDA-cleared) with point-of-care testing available (<20 min) [67]. I-FABP lacks standardized assays, and its short half-life (11 min) limits clinical utility outside research settings [81,82].

Clinical Recommendation: Given current evidence and availability, suPAR and IL-6 represent the most actionable biomarkers for risk stratification. However, no guidelines currently recommend routine biomarker-guided therapy. Their primary utility lies in identifying high-risk patients who warrant aggressive cardiovascular risk factor management and statin therapy, as supported by the REPRIEVE trial [64,83].

## 8. Mechanisms of Persistent Immune Dysfunction

### 8.1. Viral Reservoir and Residual Viremia

Multiple, interconnected mechanisms sustain immune dysfunction despite virological suppression (summarized in Table S4). The viral reservoir, established within long-lived memory CD4+ T cells harboring transcriptionally silent provirus, has an estimated half-life of approximately 44 months, precluding eradication with cART alone [3,84,85]. The reservoir contributes to inflammation through stochastic reactivation events that trigger innate immune responses, production of aberrant proteins from defective proviruses, and ongoing low-level replication in anatomical sanctuaries where antiretroviral penetration may be suboptimal [84,85,86].

### 8.2. Viral Coinfections

Chronic viral coinfections, particularly cytomegalovirus (CMV), contribute substantially to immune activation by persistently stimulating antigens and inducing T cell exhaustion [73]. CMV seropositivity is associated with expanded differentiated effector T cell populations and elevated inflammatory markers [74,75]. Successful treatment of hepatitis C virus (HCV) coinfection with direct-acting antivirals reduces sCD14 and IL-6 levels, demonstrating the contribution of viral coinfections to chronic inflammation [76,87]. Lymphoid tissue fibrosis, mediated by TGF-β-dependent collagen deposition, limits naïve T-cell repopulation and contributes to incomplete immune reconstitution [3,88,89].

### 8.3. Accelerated Biological Aging and the Geroscience Hypothesis

HIV infection accelerates epigenetic aging by approximately 5–9 years, beginning at the time of initial infection and persisting despite suppressive ART [90,91]. Studies using epigenetic clocks (Horvath, GrimAge, DunedinPACE) consistently demonstrate that PLWH on stable ART exhibit biological ages significantly older than their chronological ages, with the magnitude of acceleration correlating with nadir CD4 count and duration of untreated viremia [90,91].

The geroscience hypothesis proposes that targeting fundamental aging hallmarks—cellular senescence, mitochondrial dysfunction, and inflammaging—could simultaneously address multiple HIV-associated comorbidities rather than treating each individually [92]. This framework has led to clinical trials of senolytics (dasatinib + quercetin) in frail and prefrail PLWH, with early-phase results indicating favorable safety profiles. Notably, the REPRIEVE epigenetic aging substudy demonstrated that pitavastatin prevented acceleration of biological aging over 24 months (measured by DunedinPACE), representing the first evidence that statins may slow epigenetic aging in PLWH [93]. This finding bridges the geroscience framework with currently available therapeutics.

### 8.4. Sex Differences in HIV-Associated Inflammation

Emerging evidence highlights clinically relevant sex-based differences in immune activation, with approximately half of the 40 million PLWH globally being women [94,95]. Women living with HIV demonstrate higher type I interferon (IFN-α) production, stronger mucosal proinflammatory responses, and less reduction in immune activation markers on ART compared to men [94]. These differences are partly attributable to biallelic TLR7 expression resulting from incomplete X-chromosome inactivation, leading to enhanced plasmacytoid dendritic cell responses [94]. These findings underscore the need for sex-stratified analyses in clinical trials evaluating anti-inflammatory strategies in PLWH.

### 8.5. Myeloid Compartment Activation, Inflammasome Signaling and Cellular Senescence

Recent mechanistic studies have converged on the monocyte/macrophage compartment, inflammasome signaling, and cellular senescence as central, interconnected drivers of persistent immune dysfunction in cART-suppressed HIV infection. In a longitudinal transcriptomic study of SIV-infected rhesus macaques, Chen et al. (2026) demonstrated that short-term ART normalizes the acute interferon-stimulated gene response, but long-term ART is characterized by reactivation of TLR2/TLR4/MyD88 signaling, sustained NF-κB and NLRP3/NLRP12–caspase-1 activation, persistent macrophage activation, and a senescence-associated secretory phenotype (SASP) in peripheral blood mononuclear cells [96]. Pathological examination of carotid arteries from animals on long-term ART revealed macrophage-rich plaques infiltrated by p21+ senescent cells with intraluminal thrombus formation—a recapitulation of HIV-associated atherogenesis in humans [96]. These data provide direct in vivo evidence that the myeloid–inflammasome–senescence triad operates regardless of effective viral suppression.

In humans, complementary findings have emerged. Leal et al. described a compartment-specific dysregulation of NLRP3, with hyper-activation in circulating B lymphocytes (driving polyclonal IgM secretion and influencing the response to HBV vaccination) on the background of a relatively “exhausted” NLRP3 response in the myeloid compartment of PLWH on ART, indicating that the inflammasome contribution to chronic inflammation is not uniform across leukocyte subsets [97]. At the cellular-aging level, Li et al. (2025), within the Multicenter AIDS Cohort Study, reported that SASP markers—including matrix metalloproteinase-9, growth/differentiation factor-15, stanniocalcin-1, and SerpinE1—discriminate PLWH from controls and that MMP-9 correlates with intact HIV-1 proviral DNA, linking the senescent secretome to the size of the viral reservoir [98]. Earlier work by Lagathu and colleagues had already framed inflammaging in HIV as the convergence of cellular senescence, mitochondrial dysfunction, altered gut microbiota, and coinfections, and proposed inflammatory/innate-immunity markers as candidates for clinical follow-up [40]. CD8+ T cells acquire CD28−CD57+ senescent phenotypes that further amplify the proinflammatory milieu, a pattern shared with CMV reactivation and aging [41]. Taken together, the myeloid–inflammasome–senescence axis represents a major mechanistic gap not closed by virological suppression, and it directly informs the therapeutic strategies discussed in Section 9, including statins (which appear to slow epigenetic ageing in REPRIEVE) [93], senolytics (dasatinib + quercetin) [92], NLRP3-targeted agents currently in early-phase trials, and gut-directed interventions that reduce the upstream antigenic load.

## 9. Therapeutic Implications and Clinical Recommendations

The recognition that chronic immune activation persists despite virological suppression has prompted investigation of adjunctive anti-inflammatory strategies [80]. Table 2 summarizes the landmark clinical trials that have shaped our understanding of HIV-associated inflammation and its therapeutic modification.

### 9.1. Statins: The REPRIEVE Paradigm and Mechanistic Insights

The landmark REPRIEVE trial demonstrated that pitavastatin treatment reduced major adverse cardiovascular events (MACE) by 35% among 7769 PLWH with low-to-moderate cardiovascular risk (HR 0.65, 95% CI 0.48–0.90, *p* = 0.002) over a median follow-up of 5.1 years [83]. An updated analysis with extended follow-up (median 5.6 years, 257 events) strengthened this finding (HR 0.64, 95% CI 0.48–0.84) [64].

Notably, subsequent mechanistic substudies have substantially refined our understanding of how pitavastatin reduces cardiovascular events. Lu et al. demonstrated significant reductions in noncalcified coronary plaque volume, Lp-PLA2, and oxidized LDL, but hs-CRP reduction was not statistically significant (*p* = 0.09) [64]. This critical finding suggests that MACE reduction may operate through non-classical anti-inflammatory pathways—primarily plaque stabilization rather than systemic CRP-mediated inflammation [64]. A targeted proteomics analysis by Kolossváry et al. identified PCOLCE (procollagen C-endopeptidase enhancer 1) as the protein most strongly associated with plaque reduction, independent of LDL-C changes, pointing to extracellular matrix remodeling as a key mechanism [99]. Additionally, a pilot epigenetic aging substudy demonstrated that pitavastatin prevented acceleration of DunedinPACE over 24 months, suggesting benefits on biological aging [93]. Pitavastatin was chosen due to minimal drug–drug interactions with antiretrovirals via CYP450 [100].

Clinical Implication: These mechanistic data indicate that REPRIEVE’s cardiovascular benefits extend beyond simple LDL reduction and classical anti-inflammatory effects. The plaque stabilization, lipid modification, and potential anti-aging properties collectively support the use of statins as a multimodal intervention in PLWH at cardiovascular risk.

### 9.2. Early cART Initiation

The START trial demonstrated that immediate cART initiation (regardless of CD4 count) reduced the composite endpoint of AIDS, serious non-AIDS events, or death by 57% (HR 0.43) compared to deferral until CD4 < 350 cells/μL [101]. Early treatment preserves gut mucosal immunity, limits GALT destruction, and reduces reservoir seeding [102,103]. The TEMPRANO trial corroborated these findings in resource-limited settings [102]. However, early cART does not entirely prevent chronic immune activation, highlighting the need for adjunctive strategies.

### 9.3. Antiretroviral Selection: INSTIs vs. PIs

Integrase strand transfer inhibitors (INSTIs) demonstrate favorable inflammatory biomarker profiles compared to protease inhibitor-based regimens (Table S5) [104,105]. Our previous work demonstrated that INSTI-based regimens were associated with significantly lower IL-6 levels (5.65 vs. 7.11 pg/mL; *p* = 0.03) and better normalization of LBP (33% vs. 0% within the normal range; *p* = 0.04) compared with PI-based regimens in unadjusted analyses, although these differences were attenuated after inverse probability of treatment weighting, underscoring the need for larger confirmatory studies [104].

INSTI-Associated Weight Gain—A Double-Edged Sword: While INSTIs demonstrate favorable inflammatory profiles, the association with weight gain (particularly dolutegravir and bictegravir) has emerged as a clinical concern [106,107]. Pooled analyses suggest a median weight gain of 2.0 kg over 96 weeks overall, with higher gains observed in tenofovir alafenamide (TAF)-containing regimens, in Black individuals, and in women [106,107]. A 2025 study found that INSTI-associated weight gain was inversely correlated with the abundance of specific gut microbial taxa (*Dysosmobacter*, *Colidextribacter*), suggesting a microbiome-mediated mechanism [108]. Crucially, the DO-IT trial presented at IAS 2025 demonstrated that switching off INSTIs does not reverse weight gain, indicating that alternative strategies—including GLP-1 receptor agonists and lifestyle interventions—are needed for weight management [109]. Visceral adipose tissue is proinflammatory, secreting IL-6, TNF-α, and adipokines that may counteract the anti-inflammatory effects of viral suppression [110,111,112].

More recent mechanistic and clinical data refine, rather than contradict, this picture. The nested case–control metabolomic analysis from the MICTLAN trial showed that, in PLWH starting BIC/TAF/FTC or DTG/ABC/3TC, >10% weight gain at 18 months was driven by insulin resistance (HOMA-IR), visceral adiposity (>4 cm), and hypertriglyceridaemia, with accumulation of medium-chain acylcarnitines pointing to mitochondrial dysfunction and dysregulated lipid metabolism as proximate mechanisms [113]. In parallel, in vitro and murine work by Gisbert-Ferrándiz et al. (2025) demonstrated that DTG and BIC exert distinct but overlapping effects on adipocyte biology: DTG suppresses adipocyte differentiation and adipokine expression while up-regulating profibrotic genes, whereas BIC accelerates adipogenesis with hypertrophic adipocytes [114]. Capeau and colleagues, reviewing the most recent literature, conclude that the INSTI and TAF effects are additive, occur predominantly within the first 12 months of exposure, and show only limited reversibility on switching, with African origin, female sex, low baseline CD4 count, and high baseline HIV RNA as consistent risk factors [107]. Our previous interpretation [106,107,108,109] is therefore strengthened, not refuted, by this updated literature: switching off INSTIs is unlikely to reverse established weight gain, and management should focus on early identification of metabolic risk, lifestyle interventions, and—when appropriate—GLP-1 receptor agonists (Section 9.4), rather than on antiretroviral substitution alone [113,114].

Clinical Recommendation: ART selection should account for individual metabolic profiles and cardiovascular risk [115,116]. For patients with pre-existing obesity or metabolic syndrome, NNRTI-based alternatives (doravirine) may be considered, balanced against the superior virological efficacy and barrier to resistance of INSTIs [115,116]. Weight monitoring and lifestyle interventions are essential for all patients on INSTI-based therapy [115,116].

### 9.4. Emerging Therapies: GLP-1 Receptor Agonists

Glucagon-like peptide-1 receptor agonists (GLP-1 RAs) represent a potentially transformative therapeutic class for HIV-associated metabolic inflammation. Eckard et al. demonstrated in a phase 2b randomized, placebo-controlled trial that once-weekly semaglutide reduced visceral adipose tissue by 30.6% and significantly lowered IL-6 and sCD163 levels in PLWH with lipohypertrophy [117]. A post hoc epigenetic analysis found that semaglutide slowed biological aging by approximately 3–5 years (as measured by the PCGrimAge and PhenoAge clocks), suggesting benefits that extend beyond weight and fat reduction [118].

Given that INSTI-associated weight gain drives metabolic inflammation and is not reversible by switching antiretrovirals, GLP-1 RAs address a critical unmet need at the intersection of metabolic dysfunction and chronic inflammation [109]. Phase III trials evaluating cardiovascular outcomes of GLP-1 RAs specifically in PLWH are warranted.

Clinical Caveat—GLP-1 RAs and Antiretroviral Pharmacokinetics: GLP-1 RAs delay gastric emptying and can therefore alter the absorption of orally administered drugs with food- or pH-dependent bioavailability [119]. This is clinically relevant in PLWH because several widely used antiretrovirals fall into this category. Rilpivirine requires a substantial meal (≥390 kcal) and an acidic gastric pH for adequate absorption [120], and atazanavir-based regimens are similarly sensitive to gastric pH [121]; tenofovir alafenamide and bictegravir absorption may also be modestly affected [122]. While dedicated pharmacokinetic studies of semaglutide or other GLP-1 RAs in combination with cART are still limited, prudence dictates that GLP-1 RAs be used with caution in patients receiving rilpivirine- or atazanavir-containing regimens, with reinforced counseling on food intake, closer virological monitoring during dose escalation, and consideration of alternative cART backbones in patients with sub-optimal adherence or marginal virological control. Long-acting parenteral antiretrovirals (cabotegravir/rilpivirine LA, lenacapavir) bypass this potential interaction and may be preferable in selected patients in whom GLP-1 RAs are strongly indicated.

### 9.5. JAK Inhibitors and Targeted Anti-Inflammatory Strategies

Janus kinase (JAK) inhibitors target the inflammatory cascade upstream of specific cytokines. The ACTG A5336 trial demonstrated that ruxolitinib significantly decreased sCD14, IL-18, and markers of immune activation in ART-suppressed PLWH, with follow-up data suggesting concurrent decay of the viral reservoir [123]. By targeting the JAK–STAT signaling pathway—which mediates responses to IL-6, IFN-α, and other inflammatory cytokines central to HIV-associated inflammation—this class could simultaneously address multiple inflammatory pathways [123]. However, the risk of infection with long-term JAK inhibition necessitates careful evaluation in immunocompromised PLWH [123].

### 9.6. Comprehensive Therapeutic Strategies

**Figure 3:**
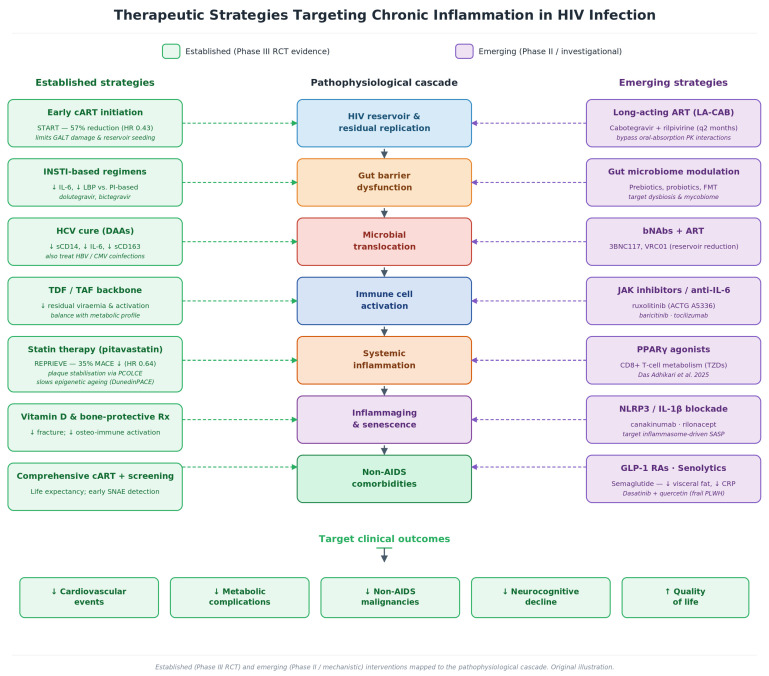
Therapeutic strategies targeting chronic inflammation in HIV infection. Evidence-based strategies (green, Phase III RCT data) and emerging interventions (purple, Phase II/observational) targeting specific components of the pathophysiological cascade from HIV reservoir and residual replication through gut barrier dysfunction, microbial translocation, immune cell activation, and systemic inflammation to non-AIDS comorbidities [47]. Green dashed arrows indicate established interventions acting on the corresponding pathophysiological step; purple dashed arrows indicate emerging interventions; vertical black arrows indicate progression along the pathophysiological cascade. Colors in the central cascade boxes are used only to differentiate sequential steps and carry no categorical meaning. ↑ increase; ↓ decrease.

Table S6 provides a comprehensive overview of current and emerging therapeutic strategies targeting chronic inflammation in HIV infection. Evidence-based interventions include early cART initiation, statin therapy, INSTI-based regimens, and treatment of viral coinfections [67,69,83,105,106]. Emerging strategies under investigation include GLP-1 receptor agonists, PPARγ agonists, JAK inhibitors, modulation of the gut microbiome, senolytics, and vedolizumab (anti-α4β7) targeting gut immune cell trafficking (Figure 3) [14,48,67,69,83,105,106,117,123].

Unanswered Questions

- Can gut barrier integrity be fully restored in PLWH who initiated cART during chronic infection, and can reversal of trained immunity contribute to this restoration?
- What is the relative contribution of viral reservoirs versus microbial translocation versus trained immunity to persistent immune activation?
- Do GLP-1 receptor agonists reduce cardiovascular events in PLWH through anti-inflammatory mechanisms beyond weight reduction?
- Can senolytic therapies reverse HIV-accelerated biological aging and reduce multimorbidity?
- How do novel ART regimens (long-acting injectables, lenacapavir) affect chronic inflammation compared to oral cART?
- Can biomarker-guided intensification strategies (suPAR/IL-6-directed statin initiation) improve clinical outcomes in high-risk PLWH?
- Do sex-specific differences in immune activation necessitate sex-stratified therapeutic approaches?

## 10. Conclusions

Chronic immune activation represents the fundamental driver of non-AIDS morbidity and mortality in the cART era, resulting in a 16-year reduction in comorbidity-free life expectancy that defines the actual burden of treated HIV infection [10]. The pathophysiological cascade from HIV-mediated GALT destruction through microbial translocation and trained immunity to systemic inflammation provides a mechanistic framework for understanding why virologically suppressed patients experience accelerated aging and multimorbidity.

Four recent advances fundamentally reshape this framework. First, the identification of CD8+ T cell immunometabolic dysfunction as a driver of epithelial barrier disruption establishes PPARγ agonists as potential therapeutic targets [47]. Second, recognition that maladaptive trained immunity in monocytes perpetuates inflammation through epigenetic reprogramming that is independent of ongoing microbial translocation identifies novel therapeutic avenues, including IL-1β pathway blockade and epigenetic modifiers [34,39]. Third, longitudinal transcriptomic, proteomic, and tissue-pathology data now demonstrate that persistent monocyte/macrophage activation, NLRP3 inflammasome signaling, and cellular senescence (SASP) form an interconnected axis that operates regardless of effective viral suppression and is directly linked to vascular and metabolic comorbidities—an axis amenable to agents that selectively eliminate senescent cells (senolytics), NLRP3-directed agents, and statins [40,41,92,93,96,97,98]. Fourth, the REPRIEVE mechanistic substudies have revealed that statin-mediated cardiovascular protection operates through plaque stabilization via PCOLCE-mediated procollagen pathways rather than classical systemic anti-inflammatory mechanisms, with additional benefits on biological aging [64,93,99].

Beyond these mechanistic gains, gut microbiome shifts deserve specific recognition within the clinical framework, not just within the pathophysiological cascade. Dysbiosis in PLWH—characterized by expansion of *Enterobacteriaceae* and *Proteobacteria*, depletion of butyrate-producing *Firmicutes* such as *Faecalibacterium prausnitzii* and *Roseburia*, and disruption of the gut mycobiome with overgrowth of *Candida* species—provides a continuous antigenic load that sustains microbial translocation, primes the maladaptive arm of innate immunity, and amplifies myeloid-driven inflammation [50,51,52,53,54,55]. The recent demonstration that dysbiotic stool from PLWH can transfer susceptibility to *Cryptosporidium parvum* in humanized-mouse models [54] supports the view that the gut microbiome is not a passive marker of HIV-related immune dysfunction but a modifiable factor in the pathway to non-AIDS comorbidities. Gut-directed strategies—prebiotics and probiotics aimed at restoring butyrate producers, fecal microbiota transplantation, selective targeting of the mycobiome, and adjunctive bacterial-product-neutralizing approaches—therefore belong alongside statins, GLP-1 receptor agonists, and senescence-targeting therapies in the clinician’s toolkit, even if most remain investigational at this time and require rigorous outcome-driven trials in PLWH.

The integration of biomarker-guided risk stratification with evidence-based therapeutic interventions—early cART, statins, optimal ART selection—and emerging strategies including GLP-1 receptor agonists, drugs that selectively eliminate senescent cells (senolytics), and JAK inhibitors offers a comprehensive approach to reducing inflammation-associated comorbidities in PLWH. Future research must address critical unanswered questions, particularly the optimal timing of anti-inflammatory interventions, the clinical utility of therapies targeting trained immunity, and the development of sex-stratified treatment algorithms. For now, the clinician’s armamentarium includes validated strategies that can meaningfully improve outcomes for the approximately 40 million PLWH worldwide [95].

Clinical Practice Points

1. Consider statin therapy (pitavastatin preferred) for all PLWH aged 40–75 based on REPRIEVE evidence; benefits extend beyond LDL reduction to include plaque stabilization and potential anti-aging effects [64,83,93,99].
2. Initiate cART as early as possible to preserve gut mucosal immunity and limit GALT destruction [102,103,104].
3. Prefer INSTI-based regimens for favorable inflammatory profiles, but monitor weight closely; if significant weight gain occurs, consider GLP-1 RAs rather than switching off INSTIs [106,107,109,117].
4. Use suPAR or IL-6 to identify high-risk patients for aggressive cardiovascular risk factor management [68,70].
5. Treat viral coinfections promptly—HCV cure with DAAs reduces sCD14 and IL-6 [76,87].
6. Screen for and manage metabolic syndrome, particularly in patients on TAF-containing regimens and those with visceral adiposity [107,110].
7. Consider sex-specific risk: women may have higher residual immune activation despite adequate viral suppression [94].

## 11. Limitations

This narrative review has several limitations. First, as this is a non-systematic review, selection bias in the inclusion of studies cannot be ruled out. However, we prioritized high-quality RCTs, large cohort studies, and mechanistic investigations published in high-impact journals and conducted the review in accordance with the SANRA checklist. Second, biomarker data derive predominantly from North American and European cohorts, limiting generalizability to resource-limited settings where HIV burden is greatest. Third, the rapidly evolving therapeutic landscape—including long-acting injectables (cabotegravir/rilpivirine) and lenacapavir—means that data on inflammation for the newest regimens remain limited. Fourth, we focused on biomarkers with established prognostic validation; emerging markers (e.g., galectin-9, soluble TRAIL) may prove clinically useful pending further validation. Fifth, trained immunity in PLWH has been primarily characterized in cross-sectional and ex vivo studies; longitudinal data confirming its causal role and responsiveness to therapeutic intervention are needed. Sixth, the gut-microbiome signatures we describe show substantial cohort-to-cohort variability and are confounded by transmission risk group and geography [54,55]; given the predominantly cross-sectional nature of the available data, a direct pathogenetic role for specific dysbiotic patterns should be inferred cautiously and ideally confirmed in longitudinal and interventional studies. Finally, although we discuss therapeutic interventions, most anti-inflammatory strategies beyond statins remain investigational, and translation to clinical practice requires completion of Phase III trials.

## Figures and Tables

**Table 1:** Biomarkers of immune activation and intestinal barrier dysfunction.

Biomarker	Source/Mechanism	Clinical Association	Effect Size	Key Studies
sCD14	Released from monocytes upon LPS stimulation	All-cause mortality; CVD events	OR 6.0 (mortality)	SMART [66]
IL-6	Proinflammatory cytokine; systemic inflammation	Mortality; CVD; frailty	HR 1.8–2.5	SMART/ESPRIT [58,59]
suPAR	Cleaved from uPAR; immune activation marker	Mortality; kidney disease; CVD	HR 2.1–3.4	ACTG A5001 [67]
I-FABP	Released from damaged enterocytes	Gut barrier damage; mortality	HR 1.5–2.0	Hunt et al. [68]
LBP	Acute phase protein; LPS binding	Microbial translocation; CVD	HR 1.4–1.8	Multiple cohorts [69,70,71]
D-dimer	Fibrin degradation product	Coagulation; mortality	HR 1.4–2.1	SMART [59]

Abbreviations: sCD14, soluble CD14; IL-6, interleukin-6; suPAR, soluble urokinase plasminogen activator receptor; I-FABP, intestinal fatty acid-binding protein; LBP, lipopolysaccharide-binding protein; CVD, cardiovascular disease; OR, odds ratio; HR, hazard ratio.

**Table 2:** Landmark clinical trials informing HIV inflammation management.

Trial	Design	Intervention	Primary Outcome	Key Finding
SMART	RCT; *n* = 5472; CD4 > 350	Continuous vs. intermittent ART	AIDS/death or major SNAEs	Interruption ↑ mortality (HR 1.8)
START	RCT; *n* = 4685; ART-naive; CD4 > 500	Immediate vs. deferred ART	AIDS, SNAEs, or death	Immediate ART: 57% ↓ events (HR 0.43)
REPRIEVE	RCT; *n* = 7769; age 40–75; low–mod CVD risk	Pitavastatin 4 mg vs. placebo	MACE	35% ↓ MACE (HR 0.65; updated HR 0.64)
ESPRIT	RCT; *n* = 4111; CD4 > 300 on ART	IL-2 + ART vs. ART alone	OI or death	No benefit; IL-6 predicts mortality
SILCAAT	RCT; *n* = 1695; CD4 50–299 on ART	IL-2 + ART vs. ART alone	OI or death	No benefit; IL-6 as prognostic marker

Abbreviations: RCT, randomized controlled trial; CD4, cluster of differentiation 4; ART, antiretroviral therapy; SNAEs, serious non-AIDS events; MACE, major adverse cardiovascular events; OI, opportunistic infection; HR, hazard ratio; ↑ increase; ↓ decrease.

## Data Availability

No new data were created or analyzed in this study. Data sharing does not apply to this article.

## Supplementary Materials

The following supporting information can be downloaded at: https://www.mdpi.com/article/10.3390/microorganisms14061229/s1, Table S1: HIV-1 Genomic Organization and Gene Products; Table S2: Intrinsic antiviral restriction factors and their viral antagonists; Table S3: Gut microbiome alterations in HIV infection; Table S4: Mechanisms contributing to chronic immune activation in treated HIV; Table S5: Comparison of INSTI-based vs. PI-based regimens on inflammatory parameters; Table S6: Therapeutic strategies targeting chronic inflammation in HIV.

## Abbreviations

AIDS	acquired immunodeficiency syndrome
APOBEC3G	apolipoprotein B mRNA-editing enzyme catalytic polypeptide-like 3G
ART	antiretroviral therapy
bNAbs	broadly neutralizing antibodies
BST-2	bone marrow stromal antigen 2 (tetherin)
cART	combined antiretroviral therapy
cGAS	cyclic GMP–AMP synthase
CMV	cytomegalovirus
CVD	cardiovascular disease
DAAs	direct-acting antivirals
D-dimer	fibrin degradation product
DNA	deoxyribonucleic acid
dNTP	deoxynucleoside triphosphate
DunedinPACE	DunedinPACE epigenetic clock
FMT	fecal microbiota transplantation
GALT	gut-associated lymphoid tissue
GLP-1 RA	glucagon-like peptide-1 receptor agonist
HBV	hepatitis B virus
HCV	hepatitis C virus
HDAC	histone deacetylase
HIV	human immunodeficiency virus
HR	hazard ratio
hs-CRP	high-sensitivity C-reactive protein
I-FABP	intestinal fatty acid-binding protein
IDO	indoleamine 2,3-dioxygenase
IFN	interferon
IL-1β	interleukin-1 beta
IL-6	interleukin-6
INSTIs	integrase strand transfer inhibitors
IRF3	interferon regulatory factor 3
JAK	Janus kinase
LBP	lipopolysaccharide-binding protein
LDL	low-density lipoprotein
LPS	lipopolysaccharide
LTR	long terminal repeat
MACE	major adverse cardiovascular events
MASLD	metabolic dysfunction-associated steatotic liver disease
mTOR	mammalian target of rapamycin
MxB	myxovirus resistance protein B
MyD88	myeloid differentiation primary response 88
NF-κB	nuclear factor kappa B
NNRTIs	non-nucleoside reverse transcriptase inhibitors
NRTIs	nucleoside reverse transcriptase inhibitors
OR	odds ratio
ox-LDL	oxidized low-density lipoprotein
PCOLCE	procollagen C-endopeptidase enhancer 1
PIs	protease inhibitors
PLWH	people living with HIV
PPARγ	peroxisome proliferator-activated receptor gamma
RCT	randomized controlled trial
RNA	ribonucleic acid
SAMHD1	SAM domain and HD domain-containing protein 1
SANRA	Scale for the Assessment of Narrative Review Articles
sCD14	soluble CD14
SCFA	short-chain fatty acid
SERINC3/5	serine incorporator 3/5
SNAEs	serious non-AIDS events
STAT	signal transducer and activator of transcription
STING	stimulator of interferon genes
suPAR	soluble urokinase plasminogen activator receptor
TAF	tenofovir alafenamide
TGF-β	transforming growth factor beta
Th17	T helper 17 cells
TLR	Toll-like receptor
TNF-α	tumor necrosis factor alpha
TRIM5α	tripartite motif-containing protein 5 alpha
TRIF	TIR domain-containing adapter-inducing interferon-β
TZDs	thiazolidinediones
uPAR	urokinase plasminogen activator receptor
