# Supplementary material for: The Gut–Immune Axis in Treated HIV Infection: From Mucosal Damage to Chronic Inflammation and Therapeutic Opportunities—A Clinician-Oriented Narrative Review
Source: Microorganisms. 2026 May 29;14(6):1229. doi: 10.3390/microorganisms14061229 (PMC13303623; doi:10.3390/microorganisms14061229)
Supplement: Supplementary file 1 [file microorganisms-14-01229-s001.zip › microorganisms-4295822-supplementary.pdf]

microorganisms

MDPI

Supplementary Materials

The Gut–Immune Axis in Treated HIV Infection: From Mucosal Damage to Chronic Inflammation and Therapeutic Opportunities—A Clinician-Oriented Narrative Review

Thomas N. Nitsotolis, Stelios F. Assimakopoulos, Maria Lagadinou, Alexia Papalexandrou, Nikolaos Krikis, Marios Kourtidis, Eirini Christaki and Haralampos Milionis

Table S1. HIV-1 Genomic Organization and Gene Products.

Gene	Protein	Function	Clinical Relevance
gag	p24, p17, p7, p6	Core structural proteins: capsid, matrix, nucleocapsid	p24: diagnostic marker; target for lenacapavir
pol	RT, PR, IN	Reverse transcriptase, protease, and integrase enzymes	Primary targets of NRTIs, NNRTIs, PIs, INSTIs
env	gp120, gp41	Envelope glycoproteins for viral entry	Target for entry inhibitors and bNAbs
tat	Tat	Transactivator of viral transcription	Contributes to neurotoxicity and inflammation
rev	Rev	RNA transport from the nucleus to the cytoplasm	Essential for viral replication
nef	Nef	CD4/MHC-I downregulation; counteracts SERINC3/5	Associated with disease progression
vif	Vif	Counteracts APOBEC3G via proteasomal degradation	Potential target for enhancing intrinsic immunity
vpr	Vpr	Nuclear import of PIC; cell cycle arrest	Role in macrophage infection
vpu	Vpu	CD4 degradation; BST-2/tetherin antagonism	Enhances viral release

Abbreviations: RT, reverse transcriptase; PR, protease; IN, integrase; NRTIs, nucleoside reverse transcriptase inhibitors; NNRTIs, non-nucleoside reverse transcriptase inhibitors; PIs, protease inhibitors; INSTIs, integrase strand transfer inhibitors; bNAbs, broadly neutralizing antibodies; PIC, pre-integration complex.

Table S2. Intrinsic antiviral restriction factors and their viral antagonists.

Factor	Mechanism of Restriction	Viral Antagonist	Clinical Significance	Ref.
APOBEC3G	Cytidine deamination causes G→A hypermutation in viral DNA	Vif-mediated proteasomal degradation	Potential therapeutic target; hypermutation as a viral evolution marker	[20,26]
SAMHD1	dNTP pool depletion blocking reverse transcription	Vpx (HIV-2/SIV); HIV-1 lacks antagonist	Explains myeloid cell restriction; Vpx-based vaccine strategies	[31,32]
BST-2/Tetherin	Physical tethering of budding virions to the cell membrane	Vpu-mediated downregulation and degradation	IFN-inducible; contributes to the inflammatory response	[21]

 https://doi.org/10.3390/microorganisms14061229

TRIM5α	Premature capsid uncoating and innate immune signaling	Capsid sequence variation	Species-specific restriction; basis for gene therapy approaches	[35]
MxB	Inhibition of nuclear import and integration	Unknown; capsid mutations confer partial resistance	IFN-α/β-inducible; contributes to innate defense	[36,37]
SERINC3/5	Incorporation into virions reduces infectivity	Nef-mediated exclusion from virions	Correlates with viral fitness and pathogenesis	[27,28]
MARCH8	Downregulation of envelope glycoprotein incorporation	Unknown	Tissue-specific expression affects viral tropism	[29]

Abbreviations: APOBEC3G, apolipoprotein B mRNA-editing enzyme catalytic polypeptide-like 3G; SAMHD1, SAM domain and HD domain-containing protein 1; BST-2, bone marrow stromal antigen 2; TRIM5α, tripartite motif-containing protein 5 alpha; MxB, myxovirus resistance protein B; SERINC, serine incorporator; IFN, interferon.

Table S3. Gut microbiome alterations in HIV infection.

Parameter	HIV-Associated Changes	Functional Consequences	Ref.
α-Diversity	Reduced species richness and evenness	Decreased metabolic redundancy; ecosystem instability	[51,52]
β-Diversity	Distinct community composition vs. HIV-negative	Altered metabolic output; reduced colonization resistance	[51–53]
Bacteroides spp.	Decreased abundance	Reduced SCFA production; impaired barrier function	[51,53]
Prevotella spp.	Increased abundance	Associated with mucosal inflammation; immune activation	[51,52]
Proteobacteria	Increased (esp. *Enterobacteriaceae*)	Source of LPS; drives systemic inflammation	[51,53,56]
SCFA Production	Decreased butyrate, acetate, and propionate	Impaired epithelial nutrition; reduced Treg induction	[51,115]

Abbreviations: SCFA, short-chain fatty acid; LPS, lipopolysaccharide; Treg, regulatory T cell.

Table S4. Mechanisms contributing to chronic immune activation in treated HIV.

Mechanism	Key Features	Therapeutic Implications
Viral Reservoir Persistence	Latently infected CD4+ T cells; tissue sanctuaries; intermittent low-level viremia	Cure strategies: latency reversal, gene editing, broadly neutralizing antibodies
Coinfections	CMV reactivation; HBV/HCV coinfection; subclinical bacterial/fungal infections	CMV-specific interventions; HBV/HCV treatment; antimicrobial prophylaxis
Gut Barrier Dysfunction	Irreversible GALT damage; epithelial tight junction disruption; microbial translocation	Probiotics; gut-targeted anti-inflammatories; early ART initiation
Microbial Translocation	LPS, flagellin, bacterial DNA in circulation; TLR activation; monocyte activation	sCD14 as monitoring biomarker; potential for LPS-neutralizing strategies
Immune Dysregulation	T cell exhaustion; Th17/Treg imbalance; inflammasome activation; trained immunity	Checkpoint inhibitors (research); IL-6/TNF-α blockade; JAK inhibitors
Metabolic Dysfunction	Adipose tissue inflammation; visceral fat accumulation; lipodystrophy	Weight-neutral ART selection; metabolic monitoring; lifestyle interventions

Abbreviations: GALT, gut-associated lymphoid tissue; CMV, cytomegalovirus; HBV, hepatitis B virus; HCV, hepatitis C virus; LPS, lipopolysaccharide; TLR, Toll-like receptor; ART, antiretroviral therapy; Treg, regulatory T cell; JAK, Janus kinase.

https://doi.org/10.3390/microorganisms14061229

Table S5. Comparison of INSTI-based vs. PI-based regimens on inflammatory parameters.

Parameter	INSTI-Based Regimens	PI-Based Regimens	Clinical Significance
IL-6 Levels	Greater reduction (15–25%)	Moderate reduction (10–15%)	Predicts cardiovascular events and mortality
sCD14 Levels	Significant decrease	Variable response	Marker of monocyte activation and microbial translocation
D-dimer	Favorable reduction	Less consistent reduction	Associated with thrombotic risk
hs-CRP	Improved reduction	Moderate improvement	General inflammatory marker; CVD predictor
Weight Changes	Weight gain (3–6 kg average)	Weight neutral or modest gain	May offset inflammatory benefits via visceral adiposity

Abbreviations: INSTI, integrase strand transfer inhibitor; PI, protease inhibitor; IL-6, interleukin-6; sCD14, soluble CD14; hs-CRP, high-sensitivity C-reactive protein; CVD, cardiovascular disease.

Table S6. Therapeutic strategies targeting chronic inflammation in HIV.

Strategy	Mechanism/Target	Evidence Level	Current Status
Statins (pitavastatin)	HMG-CoA reductase inhibition; pleiotropic anti-inflammatory; plaque stabilization via PCOLCE	Level A (REPRIEVE)	Recommended for primary CVD prevention in PLWH
Early ART Initiation	Limits reservoir size; preserves GALT; reduces immune activation	Level A (START, TEMPRANO)	Standard of care regardless of CD4 count
INSTI-based regimens	Favorable inflammatory profile; rapid viral suppression	Level B	Preferred first-line ART backbone
GLP-1 receptor agonists (semaglutide, liraglutide)	Visceral fat reduction; IL-6/sCD163 decrease; epigenetic age reversal	Level B (Phase 2b)	Under investigation; promising for metabolic inflammation
JAK inhibitors (ruxolitinib)	JAK–STAT blockade; sCD14/IL-18 reduction; potential reservoir decay	Level C (ACTG A5336)	Under investigation; infection risk concerns
Probiotics/Prebiotics (Lactobacillus, Bifidobacterium spp.); FMT; vedolizumab (anti-α4β7)	Microbiome modulation; SCFA production; barrier enhancement	Level C	Under investigation; mixed results
Anti-IL-6 agents (tocilizumab, siltuximab); NLRP3/IL-1β blockade (canakinumab, rilonacept)	Direct cytokine blockade	Level C	Early-phase trials ongoing
Senolytics (dasatinib + quercetin)	Clearance of senescent cells; targets aging hallmarks	Level C	Early-phase trials in frail PLWH

Abbreviations: PCOLCE, procollagen C-endopeptidase enhancer 1; CVD, cardiovascular disease; PLWH, people living with HIV; GALT, gut-associated lymphoid tissue; INSTI, integrase strand transfer inhibitor; ART, antiretroviral therapy; GLP-1, glucagon-like peptide-1; IL-6, interleukin-6; sCD163, soluble CD163; JAK, Janus kinase; STAT, signal transducer and activator of transcription; sCD14, soluble CD14; IL-18, interleukin-18; SCFA, short-chain fatty acid. Evidence levels: Level A = multiple RCTs or meta-analyses; Level B = single RCT or consistent observational data; Level C = limited evidence from early-phase trials or small observational studies.

https://doi.org/10.3390/microorganisms14061229
